# Science for service in a society is the success story of scientists with serenity

**DOI:** 10.6026/97320630016034

**Published:** 2020-01-25

**Authors:** Pandjassarame Kangueane, Senkuttuvan Pavithra

**Affiliations:** 1 Biomedical Informatics (P) Ltd

**Keywords:** Society, science, service, scientist, stories

## Abstract

Available data on science is constantly gleaned for gathering valuable information to create concise yet precise knowledge on specific subjects (especially, the biology oriented agriculture and biomedicine) for service in the society. These sacrifices surmounts as success stories for scientists worldwide. Data in the form of known literature plays a radical role in serving the society with improved infrastructure and facilities making associated resources available, accessible and affordable. This is possible by making known literature available and accessible for application in a modern economy dominant with features of democratic socialism. The public investment from tax payers coupled with the privately propelled commercial factions involved in the gathering, archiving and distribution of known literature data is complex in its current status quo in the context of creating a harmonious society with optimal social benefits. It should be noted that the role played by indirect, unorganized, unskilled and unaccounted farmers, farm laborers and service men in different layers of the supply chain in accordance with demand and supply is highly imperative. Therefore, it is of interest for a comprehensive discussion on issues in making known literature available and accessible for application towards the benefit of the society through open access publishing models within the acceptable ethical frameworks of Creative Commons (CC) and Committee on Publication Ethics (COPE).

## Description: 

**Figure 1:**
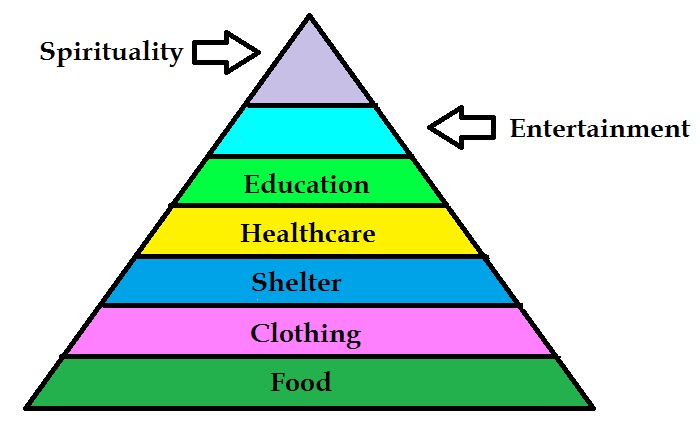
Essential needs of the society in different layers represented with varying area corresponding to the parameters.

**Figure 2:**
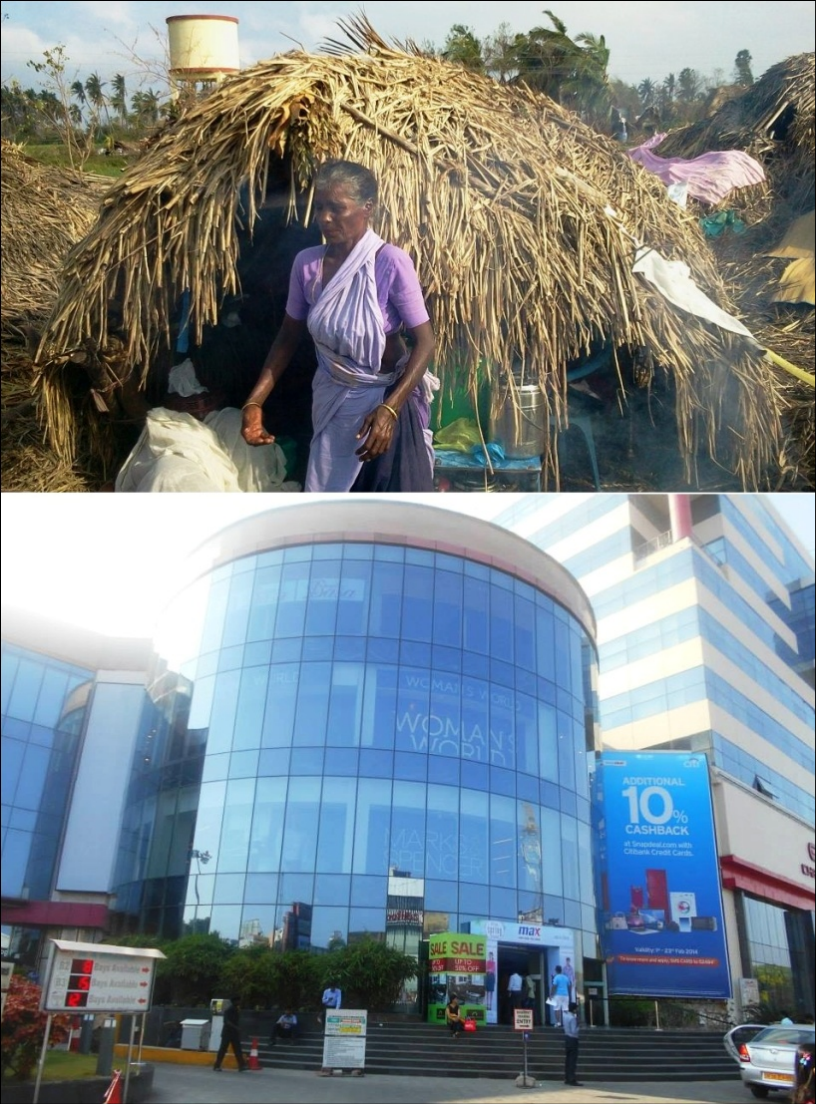
Reflection of poverty (top) and prosperity (bottom) at Tamilnadu, India

The world is passing through a difficult crossroad with multiple issues of concern with heated debates on Inflation (price rise), Fiscal (financial) deficit (shortage) and import dependency world-wide [1]. We need food in surplus, clothing, shelter, healthcare, education and entertainment towards a spiritual life (Figure 1). These essential resources could be achieved by advancing and applying known science [2]. We are trying to take services and products to the international market with pride and respect where inequality is common (Figure 2). This will create a situation where exports balance imports by strengthening equality, freedom and liberty in a global scenario under the provisions of the United Nations [3]. Therefore, the role of technology for socio-economic development is critical through strong science and established knowledge. This is possible through learning, understanding and adding knowledge to known scientific information.

The access to known scientific literature through available resources such as the products of Routledge [4], Springer [5], Elsevier [6], Taylor and Francis [7], Peter Lang [8], Sage [9], Wiley-Blackwell [10], SciELO [11], Cambridge University Press [12], Oxford University Press [13], Clarivate Analytics Inc. [14], Hindawi [15], Frontiers [16], EBSCO [17] Biomedical Informatics (P) Ltd [18], PLOS [19], World Scientific [20], AAAS [21], New Scientist Ltd. [22], Cell Press [23], NAS [24] and several others is essential (Table 1). There is a commercial aspect in making this service available to the affordable elite students and scholars (Table 1). It should be noted that revenue data for these companies is taken from elsewhere [25] as on January 25, 2020. These resources are often expensive and their access is restricted to premier institutions worldwide. This should be made available worldwide through open access models as summarized by DOAJ [26]. The US health department promotes open access databases and literature resources such as NCBI [27], NLM [28], PubMed [29], Medline [30] and PubMed Central (PMC) [31] for free over the Internet. Japan maintains the DDBJ database [32] and Europe supports the EBI/EMBL databases [33]. Many such initiatives have to be encouraged in other regions of the world including South America, Asia, Australia and Africa. The availability, accessibility and applicability of these resources are indispensable to modern economy. Hence, there is a need for more participation from the growing youth for the advancement of science to develop engineering and technology with improved precision for application in all areas of the society especially, in agriculture and healthcare. Our current challenges are unique, complex and multi-faceted in nature. These were not dealt by us in the past. We have to create novel ideas and procedures with upheld values that suit our current requirements by taking appropriate lessons where available and required. We live in a multi-lingual globe where contributions through every language have to be acknowledged and nurtured and encouraged with the help of a common binding language using enriched scientific data with an international perspective. We are all good in something. We have to give and take resources and associated data in a just and fair manner by up-holding international justice. Access to available literature for advancement through the application of science for the society is secretly sensitive yet sacred. The quote from BOAI "the promise was that removing access barriers would allow the world to "accelerate research, enrich education, share the learning of the rich with the poor and the poor with the rich … and lay the foundation for uniting humanity in a common intellectual conversation and quest for knowledge" explains everything [34]. Thus, the formation of several several open access (free to read) journals through the initiatives like PMC is engaging, entertaining and enlightening for everyone [31].

**Figure 3:**
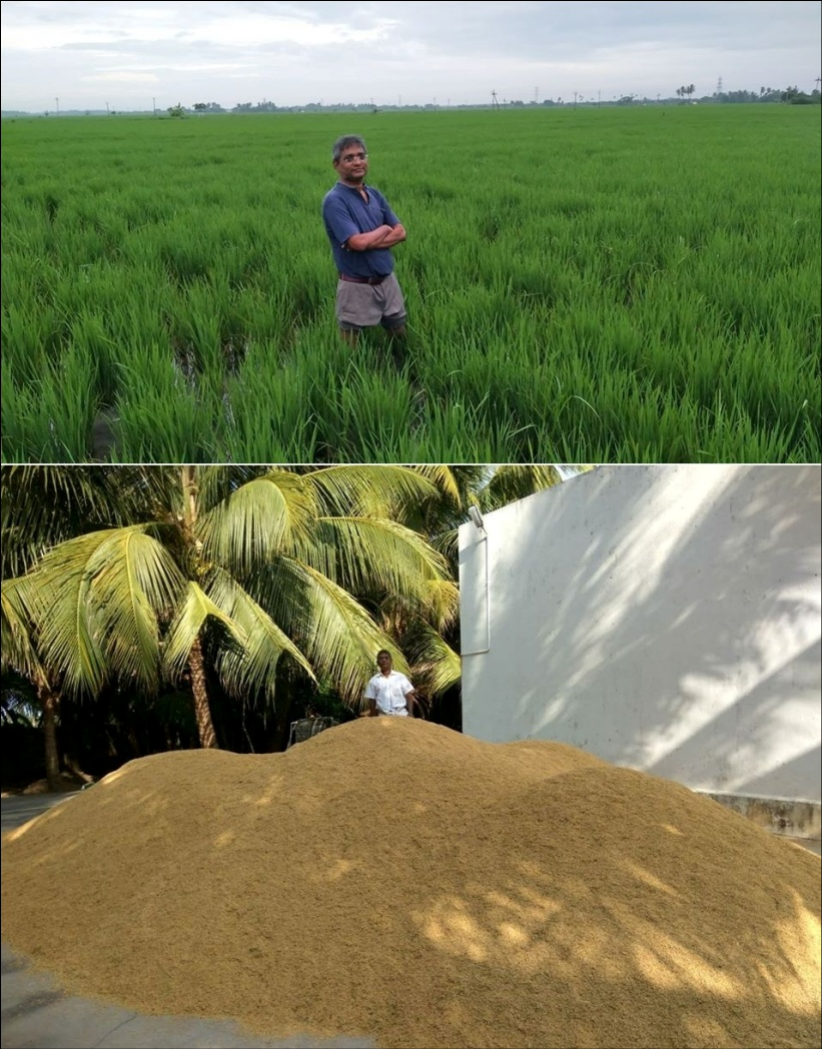
Carbohydrate rich paddy cultivation to harvest. System of rice intensification (SRI) during growth (Top); Heap of paddy produce stored after harvest (Bottom).

**Figure 4:**
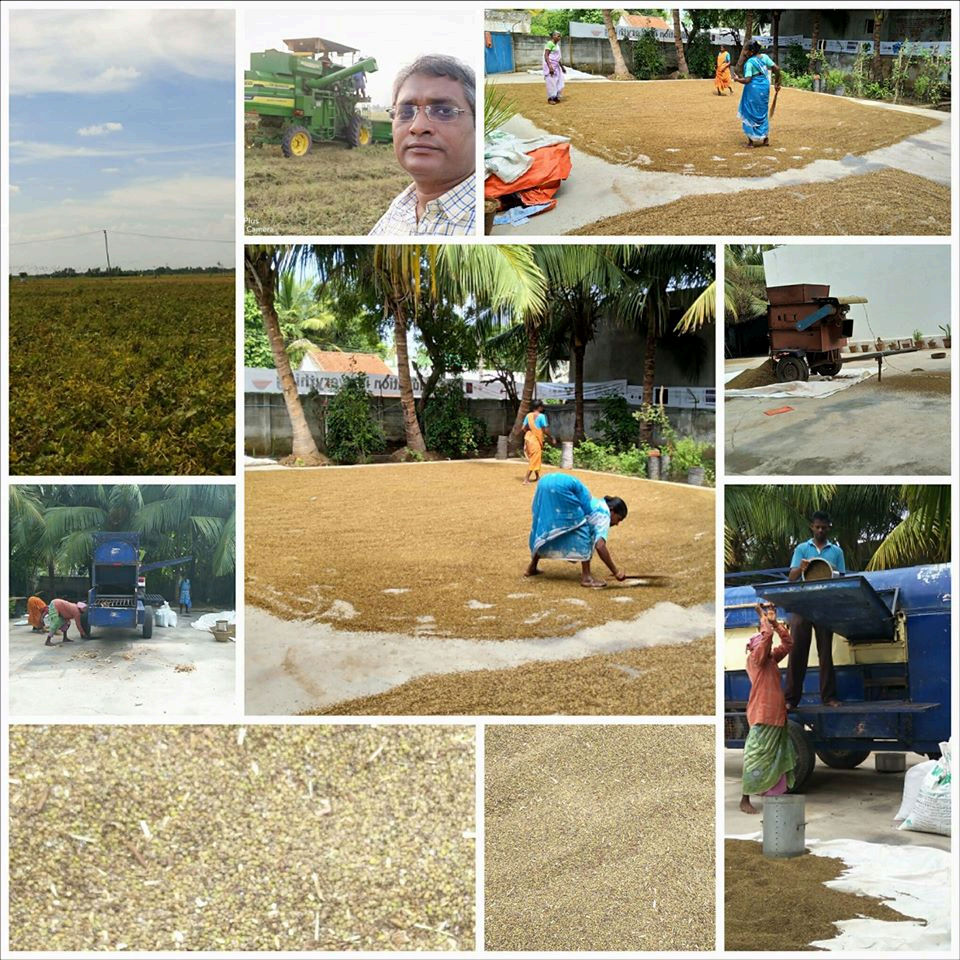
Cultivation to processing of protein rich green grams

The biology-oriented agriculture (Figure 3 and Figure 4), food processing and health care heavily depend on scientific literature in a sensitive manner. This is because concepts of mathematics, physics and chemistry are often utilized to understand complex issues in biology with an inter-disciplinary approach. Thus, science is important for everyone in daily life through an organized administrative structure. Hence, we need increased revenue receipt for spending in science, technology, agriculture and healthcare on par with international standards and benchmarks. The participation of every youth in an informed administration is vital. This is important for developing a happy society with an enduring harmony. John Adams declared the constitution and Thomas Jefferson wrote the constitution. Our era benefited freedom, equality and liberty from democracy without serious conflicts and war with the blessings of the Almighty. Honesty, hard work and perseverance served as slogans for success for billions of people around the world. We came with nothing to get something and go with nothing at the end. This is life from beginning to ending. However, the quote from Jawaharlal Nehru [35] "… but as long as there are tears and suffering, so long our work will not be over." remains as our duty until the time we pass the burning torch to the next generation willing to hold it strong for peace and prosperity.

## Conclusion:

It is of interest for a comprehensive discussion on issues to make known literature available and accessible for social application through open access publishing models within the ethical guidelines of Creative Commons (CC) [36] and Committee on Publication Ethics (COPE) [37]. Hence, we highlight data related to these issues with improved essential resources in a representative manner for societies across the world for eradicating poverty by upholding freedom, equality and liberty with natural justice.

## About the authors:

**Figure 5:**
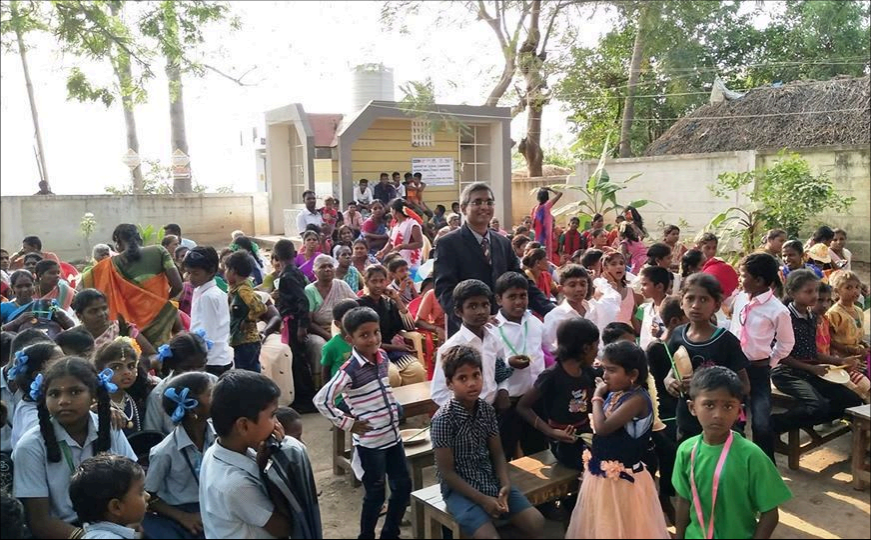
The author Pandjassarame Kangueane with the students at a public school (primary level) near a village named Irulan Sandy, Bahour Commune, Union Territory of Pondicherry, India 607 402.

Pandjassarame Kangueane (Figure 5) is a scientist, an author of scholarly materials, teacher of higher education in Biotechnology and Bioinformatics, Professor, educationalist, editor, journalist, entrepreneur, social reformer, and a farmer. He hopes to bring smile in the face of the under-privileged in every possible way. He received his Bachelor of Technology in Industrial Biotechnology from Anna University (1997), India and Doctor of Philosophy in Bioinformatics from the National University of Singapore (2001), Singapore. He served as scientist, S*Bio Pte Ltd, Singapore (2001), visiting scientist, Chiron Corporation Inc, Emeryville, California, USA (2001), Assistant Professor, Nanyang Technological University, Singapore (2002-2006), Director, Biomedical Informatics Private Ltd, India (2001-till date), Visiting Professor, VIT University, India (2007-2009), Professor, AIMST University, Malaysia (2009-2011), Chief Editor, Bioinformation, India (2005-till date) and Associate Editor, BMC Bioinformatics, Biomed Central, United Kingdom (2007-till date). He is an active farmer cultivating paddy, sugarcane, black grams, green grams, coconuts and vegetables.

Senkuttuvan Pavithra received her Bachelor of Commerce (2016) and Master of Commerce (2018) from Pondicherry University, Union Territory of Pondicherry (a French colony of pre independence India), India. She is an alumni of the alma mater Saradha Gangadharan College, Pondicherry 600 5004 which is affiliated with Pondicherry University. She serves as production director at Biomedical Informatics (P) Ltd since 2018.

## Figures and Tables

**Table 1:** Data on major arts and science publisher

S. No	Publisher Name	Location	Year*	Revenue data [25]
1	Routledge [4,7]	UK	1836	
	Taylor and Francis [7]	UK	1852	£530 million (2017)
2	Springer [5]	Germany	1842	
	Springer Nature	UK, Germany	2015	€1.64 billion (2017)
	Biomed Central	UK	2000	
3	Elsevier [6]	Netherlands	1880	£2.54 billion (2018)
	Cell Press [23]	USA	1986	
4	Peter Lang [8]	Switzerland	1970	
5	Sage [9]	USA	1965	
6	Wiley-Blackwell [10]	USA	1922	
7	SciELO [11]	Brazil	1997	
8	Cambridge University Press [12]	UK	1534	£327 million (2019)
9	Oxford University Press [13]	UK	1586	
10	Clarivate Analytics Inc. [14]	USA	2016	US$3.55 billion (2019)
	Thomson Reuters Corporation	USA	2008	US$5.50 billion (2018)
	Thomson Corporation	USA	1989	US$6.641 billion (2006)
	Reuters group plc	USA	1851	
11	Hindawi [15]	Egypt	1997	
12	Frontiers [16]	Switzerland	2007	
13	EBSCO [17]	USA	1944	
14	Biomedical Informatics (P) Ltd [18]	India	2001	
15	PLOS [19]	USA	2000	
16	World Scientific [20]	Singapore	1981	
17	AAAS (Science) [21]	USA	1880	
18	New Scientist Ltd. [22]	UK	1956	
19	National Academy of Sciences [23]	USA	1915
